# Gender and melanoma subtype‐based prognostic implications of *MUC16* and *TTN* co‐occurrent mutations in melanoma: A retrospective multi‐study analysis

**DOI:** 10.1002/cam4.70199

**Published:** 2024-09-06

**Authors:** Nilesh Kodali, Simona Alomary, Abhijit Bhattaru, Ahmed Eldaboush, Robert A. Schwartz, Shari R. Lipner

**Affiliations:** ^1^ Rutgers New Jersey Medical School Newark New Jersey USA; ^2^ Department of Dermatology Perelman School of Medicine, University of Pennsylvania Philadelphia Pennsylvania USA; ^3^ Department of Dermatology Weill Cornell Medicine New York New York USA

**Keywords:** demographics, melanoma, MUC16, survival outcomes, TTN

## Abstract

**Background:**

Most primary cutaneous melanomas have pathogenesis driven by ultraviolet exposure and genetic mutations, whereas acral lentiginous melanoma (ALM) and metastatic melanoma are much less, if at all, linked with the former. Thus, we evaluated both ultraviolet related and non‐ultraviolet related melanomas. Mutations in the *MUC16* and *TTN* genes commonly occur concurrently in these melanoma patients, but their combined prognostic significance stratified by gender and cancer subtype remains unclear.

**Methods:**

The cBioPortal database was queried for melanoma studies and returned 16 independent studies. Data from 2447 melanoma patients were utilized including those with ALM, cutaneous melanoma (CM), and melanoma of unknown primary (MUP). Patients were grouped based on the presence or absence of *MUC16* and *TTN* mutations. Univariate Cox regression and Student's *t*‐tests were used to analyze hazard ratios and total mutation count comparisons, respectively.

**Results:**

*TTN* mutations, either alone or concurrently with *MUC16* mutations, significantly correlated with worse prognosis overall, in both genders, and in CM patients. ALM patients with both mutations had better prognoses than CM patients, while ALM patients with neither mutation had worse prognosis than CM patients. For MUP patients, only *MUC16* mutations correlated with worse prognosis. ALM patients with neither *MUC16* nor *TTN* mutations had significantly more total mutations than MUP patients, followed by CM patients.

**Conclusion:**

*TTN* mutations are a potential marker of poor prognosis in melanoma, which is amplified in the presence of concurrent *MUC16* mutations. ALM patients with neither gene mutations had worse prognosis, suggesting a protective effect of having both *MUC16* and *TTN* mutations. Only *MUC16* mutations conferred a worse prognosis for MUP patients. Comprehensive genetic profiling in melanoma patients may facilitate personalized treatment strategies to optimize patient outcomes.

## 1 INTRODUCTION

Melanoma, a malignant tumor resulting from the transformation of melanocytes, has the highest mortality compared to other relatively common primary skin cancers, with 2.1 deaths per 100,000 Americans per year. Their pathogenesis, although multi‐faceted, is largely attributed to significant ultraviolet (UV) exposure for most primary cutaneous melanomas with the except of acral lentiginous melanoma (ALM); almost all melanomas are seemingly linked with an accumulation of mutated genetic drivers.^1^ Commonly mutated genes include *BRAF*, *NRAS*, *NF1*, *CDKN2A*, *MUC16*, and *TTN*.^2^, ^3^

While some genes manifest mutations that are mutually exclusive, gene mutations in *MUC16* and *TTN* may co‐occur in a substantial proportion of patients.^4^ The clinical implications of these co‐occurrent mutations on patient prognosis are not well understood. Furthermore, gender and specific melanoma subtype may also impact prognosis. Stratification of gender and cancer subtype is critical to develop a better understanding of patient outcomes based on their genetic and demographic profiles. Therefore, we investigated the impact the two most commonly co‐occurrent genetic mutations, *MUC16* and *TTN*, on patient prognosis and the impact of gender and melanoma subtype on patient mortality risk.

## 2 METHODS

We queried the cBioPortal database for melanoma studies, utilizing mutational and patient profile data from 16 studies.^5^, ^6^, ^7^, ^8^, ^9^, ^10^, ^11^, ^12^, ^13^, ^14^, ^15^, ^16^, ^17^, ^18^, ^19^, ^20^ The cBioPortal database is an open‐access resource that contains multiple cancer genomic data sets inputted from prior cancer studies. Patients were included if they had ALM, cutaneous melanoma (CM), or melanoma of an unknown primary origin (MUP). MUP is defined as having histologically confirmed melanoma metastasis without evidence of a melanoma of primary origin. We analyzed extrapolated data for mutation count, mutated gene, gender, melanoma subtype, survival status, and overall survival (in months). Patients were grouped into four gene groups: patients with neither *MUC16* or *TTN* mutations, patients with *MUC16* mutations but no *TTN* mutations, patients with *TTN* mutations but no *MUC16* mutations, and patients with both *MUC16* and *TTN* mutations.

Mutation frequency distribution analysis and co‐occurrence analysis were conducted to rank the most frequent mutations and the most prevalent co‐occurrent mutations. Univariate cox regression hazard ratio analysis was used to compare risk of mortality based on melanoma mutation type, gender, and melanoma type both within and across gene groups. Student's *t*‐tests were used to compare the mean number of mutations between genders based on mutation type. Analysis of variance tests were used to compare the number of mutations across melanoma types based on melanoma mutation type. An *ɑ* = 0.05 was used for all analyses. Statistics were conducted in R (version 4.3, R Core Team 2023).

## 3 RESULTS

A total of 2447 melanoma patients and 2495 patient samples were included. Using mutation frequency distribution analysis, *MUC16* (62.90%) and *TTN* (61.10%) mutations were the most frequent mutations in the cohort (Table S1). Using co‐occurrence analysis of the top 50 most frequently mutated genes, *MUC16* and *TTN* were the most common co‐occurrent mutated genes occurring in 741 patient samples (OR >3; *p* < 0.001) (Table S2). Overall, 126 patients had *MUC16* mutations alone, 204 patients had *TTN* mutations alone, 765 patients had both *MUC16* and *TTN* mutations, and 1352 patients had neither *MUC16* nor *TTN* mutations.

### 3.1 Overall survival

Overall survival was compared in a pairwise manner amongst the four gene groups and hazard ratios (HR) were utilized to analyze mortality risk. Compared to patients without *MUC16* or *TTN* mutations, patients with *TTN* mutations alone (HR: 1.52; CI: 1.25–1.85; *p* < 0.001) or patients with both *MUC16* and *TTN* mutations (HR: 1.36; CI: 1.20–1.55; *p* < 0.001) had significantly worse prognosis. All other pairwise comparisons of overall survival were not significant (Table 1).

**TABLE 1:** Pairwise comparisons of overall survival across gene groups.

Gene A (reference)	Gene B	Hazard ratio	Standard error	95% confidence interval	*p*‐value
Neither	MUC16	1.14	0.133	0.879–1.48	0.322
Neither	TTN	1.52	0.100	1.25–1.85	**<0.001**
Neither	Both	1.36	0.0654	1.20–1.55	**<0.001**
MUC16	TTN	1.33	0.152	0.988–1.79	0.0597
MUC16	Both	1.17	0.131	0.907–1.51	0.225
TTN	Both	0.878	0.0957	0.728–1.06	0.175

*Note*: “Neither” refers to patients without either a *MUC16* or *TTN* mutation. “Both” refers to patients with both *MUC16* and *TTN* mutations.

Bold values are the statistically significant *p* ‐values < 0.05.

### 3.2 Gender, cancer type, and gene group interactions in overall survival

Overall survival was stratified by gender and different melanoma cancer subtypes both within and across different gene groups. In patients with neither *MUC16* or *TTN* mutations, ALM patients (HR: 1.92; CI: 1.05–3.52; *p* = 0.0352) or MUP patients (HR: 2.25; CI: 1.82–2.77; *p* < 0.001) had worse prognosis than CM patients. ALM patients harboring both *MUC16* and *TTN* mutations had better prognosis (HR: 0.268; CI: 0.111–0.648; *p* = 0.00347) than CM patients who also had both mutations. MUP patients with only *MUC16* mutations and no *TTN* mutations had poorer prognosis (HR: 4.18; CI: 2.30–7.60; *p* < 0.001) than CM patients with only *MUC16* mutations (Table 2). All other overall survival comparisons within the four gene groups were not significant.

**TABLE 2:** Gender and cancer subtype‐specific overall survival within Gene Groups.

Gene group	Hazard ratio	Standard error	95% confidence interval	*p*‐value
*Neither*				
Gender				
Female	Reference			
Male	1.6	0.109	0.938–1.44	0.172
Cancer type				
Cutaneous melanoma	Reference			
Acral lentiginous melanoma	1.92	0.310	1.05–3.52	**0.0352**
Melanoma of unknown primary	2.25	0.107	1.82–2.77	**<0.001**
*Both*				
Gender				
Female	Reference			
Male	1.16	0.0919	0.966–1.38	0.113
Cancer type				
Cutaneous melanoma	Reference			
Acral lentiginous melanoma	0.268	0.450	0.111–0.648	**0.00347**
Melanoma of unknown Primary	0.901	0.0974	0.744–1.09	0.283
*MUC16*				
Gender				
Female	Reference			
Male	1.27	0.262	0.760–2.12	**0.362**
Cancer type				
Cutaneous melanoma	Reference			
Acral lentiginous melanoma	2.01	0.394	0.929–4.36	0.0761
Melanoma of unknown primary	4.18	0.305	2.30–7.60	**<0.001**
*TTN*				
Gender				
Female	Reference			
Male	0.985	0.179	0.694–1.40	0.934
Cancer type				
Cutaneous melanoma	Reference			
Acral lentiginous melanoma	1.28	0.511	0.470–3.48	0.629
Melanoma of unknown primary	1.62	0.247	0.999–2.63	0.0506

*Note*: “Neither” refers to patients without either a *MUC16* or *TTN* mutation. “Both” refers to patients with both *MUC16* and *TTN* mutations.

Bold values are the statistically significant *p* ‐values < 0.05.

Female patients with *TTN* mutations alone (HR: 1.66; CI: 1.20–2.30; *p* = 0.00215) or both *TTN* and *MUC16* mutations (HR: 1.30; CI: 1.03–1.64; *p* = 0.0295) had worse prognosis than female patients without *MUC16* or *TTN* mutations. Male patients with only *TTN* mutations (HR: 1.46; CI: 1.13–1.87; *p* = 0.00314) or both *MUC16* and *TTN* mutations (HR: 1.35; CI: 1.16–1.58; *p* < 0.001) had poorer overall survival than males without *MUC16* or *TTN* mutations (Table 3).

**TABLE 3:** Gender and cancer subtype‐specific overall survival across gene groups.

Gene group	Hazard ratio	Standard error	95% confidence interval	*p*‐value
Gender				
Female				
Neither	Reference			
Both	1.30	0.120	1.03–1.64	**0.0295**
MUC16	1.14	0.205	0.764–1.71	0.517
TTN	1.66	0.165	1.20–2.30	**0.00215**
Male				
Neither	Reference			
Both	1.35	0.0783	1.16–1.58	**<0.001**
MUC16	1.37	0.174	0.977–1.93	0.0681
TTN	1.46	0.127	1.13–1.87	**0.00314**
Cancer type				
Cutaneous melanoma				
Neither	Reference			
Both	1.83	0.0816	1.56–2.14	**<0.001**
MUC16	0.998	0.174	0.710–1.40	0.989
TTN	1.84	0.117	1.46–2.31	**<0.001**
Acral lentiginous melanoma				
Neither	Reference			
Both	0.233	0.559	0.0779–0.697	**0.0092**
MUC16	1.03	0.474	0.408–2.61	0.946
TTN	1.44	0.592	0.451–4.60	0.537
Melanoma of unknown primary				
Neither	Reference			
Both	0.821	0.122	0.647–1.04	0.104
MUC16	2.16	0.242	1.35–3.48	**0.00143**
TTN	1.28	0.232	0.810–2.01	0.293

*Note*: “Neither” refers to patients without either a *MUC16* or *TTN* mutation. “Both” refers to patients with both *MUC16* and *TTN* mutations.

Bold values are the statistically significant *p* ‐values < 0.05.

Compared to CM patients without *MUC16* or *TTN* mutations, CM patients with either *TTN* mutations alone (HR: 1.84; CI: 1.46–2.31; *p* < 0.001) or both *MUC16* and *TTN* mutations (HR: 1.83; CI: 1.56–2.14; *p* < 0.001) had worse prognosis. ALM patients with both *MUC16* and *TTN* mutations had better prognosis (HR: 0.233; CI: 0.0779–0.697; *p* = 0.0092) than ALM patients with neither *MUC16* nor *TTN* mutations. MUP patients with *MUC16* mutations alone had increased mortality risk (HR: 2.16; CI: 1.35–3.48; *p* = 0.00143) compared to MUP patients with neither *MUC16* or *TTN* mutations. All other pairwise comparisons of overall survival across gene groups were not significant.

### 3.3 Total mutation count

In patients with neither *MUC16* or *TTN* mutations (41 **±** 51 vs. 34 **±** 40; *p* = 0.03458), patients with both *MUC16* and *TTN* mutations (2884 **±** 4152 vs. 1599 **±** 1738; *p* < 0.001), patients with *TTN* mutations alone (382 **±** 226 vs. 197 **±** 138; *p* < 0.001), and the total cohort (1696 ± 3418 vs. 803 **±** 1410; *p* < 0.001), males had greater numbers of total mutations than females. Within the total cohort, MUP patients had the greatest numbers of total mutations, followed by CM patients, and then ALM patients (1634 **±** 2493 vs. 1343 **±** 3156 vs. 860 **±** 1092; *p* = 0.0196). Amongst those with neither *MUC16* or *TTN* mutations, ALM patients had the greatest number of total mutations, followed by MUP patients and then CM patients (61 **±** 45 vs. 47 **±** 54 vs. 34 **±** 44; *p* < 0.001) (Table 4). All other comparisons of total mutation counts across gender, cancer types, and gene groups were not significant.

**TABLE 4:** Total mutation count across gender, cancer subtype, and gene group.

Gene Group	Gender					Cancer type						
	Male		Female		*p*‐value	CM		ALM		MUP		*p*‐value
	M	SD	M	SD		M	SD	M	SD	M	SD	
Total Cohort	1696	3418	803	1410	**<0.001**	1343	3156	860	1092	1634	2493	**0.0196**
Neither	41	51	34	40	**0.03458**	34	44	61	45	47	54	**<0.001**
Both	2884	4152	1599	1738	**<0.001**	2477	4061	1957	951	2668	2789	0.467
MUC16	239	114	239	183	0.9887	235	132	252	163	250	190	0.845
TTN	382	226	197	138	**<0.001**	314	216	151	169	314	218	0.143

*Note*: “Neither” refers to patients without either a *MUC16* or *TTN* mutation. “Both” refers to patients with both *MUC16* and *TTN* mutations.

Abbreviations: ALM, acral lentiginous melanoma; CM, cutaneous melanoma; M, mean; MUP, melanoma of unknown primary; SD, standard deviation.

Bold values are the statistically significant *p* ‐values < 0.05.

## 4 DISCUSSION

*TTN* and *MUC16* mutations were the most frequently mutated genes within this melanoma patient cohort and were the most common combination of concurrently mutated genes overall. These genes may be more prone to mutations because of their length. The *TTN* gene has one of the longest exon genomes (chromosome 2q31, 364 exons), and also has the greatest number of mutation sites.^21^ The *MUC16* gene has the second longest exon in the genome (chromosome 19p13, 84 exons), and is associated with increased tumor mutational burden.^22^

We found that melanoma patients with *TTN* mutations alone and melanoma patients with both *MUC16* and *TTN* mutations had worse prognosis than patients with neither *MUC16* or *TTN* mutations. However, patients with *MUC16* mutations alone had a similar prognosis to those with neither *MUC16* or *TTN* mutations. Therefore, our findings suggest that, although *MUC16* and *TTN* mutations often occur concurrently, *TTN* mutations may be the primary driver of poor prognosis in melanoma patients. We found that *TTN* mutations conferred a poorer prognosis amongst both males and females and in CM patients than patients with neither *MUC16* or *TTN* mutations. Notably, *TTN* encodes the protein titin, which interacts with 170 different protein ligands, including telethonin, actinin, sAnk1, filamin C, nebulin, tropomyosin, B‐crystallin, FHL1, FHL2, calpains 1 and 3, and muscle ankyrin repeat proteins (MARPs),^23^ influencing key cellular processes, including phosphorylation, calcium binding, and myosin binding, and potentially contributing to tumorigenesis. Titin also plays a crucial role in myofibrillar signal transduction pathways^24^ by coordinating and integrating gene activation, protein folding, quality control, and degradation,^23^ which might foster tumor growth through uncontrolled cell proliferation and reduced apoptosis.

We found that patients with both *MUC16* and *TTN* mutations had higher mortality risk than patients with neither mutation, and that patients with *TTN* mutations alone had an even higher mortality risk than patients with neither mutation, which differs from previous cancer literature. In a retrospective study analyzing somatic mutations in 480 patients with lung squamous cell carcinomas, patients with *TTN* mutations had a better prognosis than patients without *TTN* mutations.^21^ In a separate retrospective study analyzing the same 480 patient cohort, patients with *TTN* mutations had better response to immune checkpoint blockade immunotherapy than patients without *TTN* mutations.^25^ These disparities in the prognostic impact of *TTN* could be attributed to different tumorigenic pathways being active in different tumor types.

Our findings indicate a complex interplay between *MUC16* and *TTN* mutations and their impact on prognosis in ALM and CM patients. We found that ALM patients without *MUC16* and *TTN* mutations had worse prognosis compared to CM patients without these mutations. However, when both mutations were present, ALM patients had a more favorable prognosis than either CM patients with both mutations or ALM patients with neither mutation. Taken together, since presence of both *MUC16* and *TTN* mutations, particularly in ALM patients, is associated with improved overall survival, these two mutations could have an additive protective effect in ALM patients. Our findings align with a retrospective prognostic analysis of 437 samples from patients with gastric cancer, showing that presence of *MUC16* mutations were associated with better prognosis when compared to patients without *MUC16* mutations.^22^ Similarly, in another retrospective genomic analysis of 37 different solid tumors from 7 public clinical cohorts, the presence of *TTN* mutations were associated with better response to immune checkpoint blockade therapies compared to patients without *TTN* mutations.^25^

The observed improved survival of ALM patients with concurrent *MUC16* and *TTN* mutations might be attributed to the trend towards lower total mutation count in ALM patients with both mutations (in comparison to CM and MUP patients with both mutations), and the significantly higher total mutation count in ALM patients with neither mutations (in comparison to CM and MUP patients with neither mutations). Similarly, in a retrospective analysis of 262 gastric cancer patients, higher total mutation count (tumor mutational burden [TMB] ≥ 8) was associated with poorer prognosis.^26^ In addition, in a retrospective database analysis of hepatocellular carcinoma patients, increased tumor mutational burden (top 20% TMB in patient cohort) was associated with worse survival rates.^27^

In *TTN*‐mutated patients, *MUC16*‐mutated patients, and patients with neither *MUC16* nor *TTN* mutations, MUP patients consistently had worse prognosis than CM patients. Similarly, in a prospective institutional study of 2930 melanoma patients, MUP patients treated with systemic therapies had poorer outcomes than patients with melanomas of known primaries (including CM or ALM) treated with systemic therapies.^28^ For MUP patients in our study, only *MUC16* mutations correlated with overall lower survival compared to MUP patients with neither mutation. Therefore, theoretically, genetic or immunologic targeting therapy towards *MUC16* genes or *MUC16*‐associated proteins might improve overall patient outcomes for MUP patients.

Our data shows that while, on average, male melanoma patients had greater numbers of total mutations than female melanoma patients across different gene groups, there were no prognostic differences based on gender. These findings are in contrast to prior studies. For example, in a genome‐wide review of gender based differences in cancer prognosis, including melanoma, as well as lung adenocarcinoma, melanoma, urothelial cell, papillary renal cell, and hepatocellular carcinoma, males compared to females had higher mutation burden, which conferred worse overall prognosis.^29^ In this same study, female melanoma patients with high mutation counts (greater than 130 mutations) still had greater overall survival compared to males with high or low mutation counts.^29^ While we did not find gender‐based differences in survival based on mutation count, there may be gender‐specific differences in other mutually‐exclusive genetic mutations (i.e., *TP53*), which were not measured in our study. For example, in a retrospective genomic analysis using The Cancer Genome Atlas Program database, male melanoma patients harbored more *TP53* gene mutations than female melanoma patients (*TP53* mutation frequency per 100,000 people for males vs. females: 4.7 vs. 2.2; *p* ≈ 0), which was associated with poorer prognosis.^30^ In a prospective study of 3324 melanoma patients, male patients had higher prevalence of tumor ulceration (63.8% vs. 36.2%; *p* < 0.0001) and metastasis to the axial skeleton (69.3% vs. 30.7%; *p* < 0.0001) than female patients, both being independent predictors of poorer outcomes in these patients.^31^

Limitations include retrospective design, lack of data pertaining to treatment history, lifestyle and environmental factors, Breslow depth, race, ethnicity, socio‐economic factors, family history, and epigenetic background. Not all gene mutations involved in melanoma development were included in the database. Furthermore, the database may have inherent patient selection biases due to the limited set of studies included, potentially limiting the generalizability of our findings. The representation of specific institutions or geographic regions could also affect the applicability of the results to a broader population. Additionally, there is a lack of literature on how different treatment modalities might influence patient outcomes specifically for those with MUC16 and TTN mutations, which we recommend as an important area for future research.

## 5 CONCLUSION

Our study showed that *MUC16* and *TTN* were the most common co‐occurrent mutations in this cohort of melanoma patients. *TTN* mutations were a consistent marker of poor prognosis, either alone or combined with *MUC16* mutations in the overall patient cohort, CM patients, and for both genders. For ALM patients, having both *MUC16* and *TTN* mutations had a better prognosis, while having neither mutation conferred a worse prognosis. MUP patients consistently exhibited a worse prognosis than CM patients across *MUC16*‐mutated patients, *TTN*‐mutated patients, and patients with neither mutation. Only the presence of *MUC16* mutations in MUP patients was associated with a worse outcome. There were no significant gender‐based differences in mortality risk across all gene combinations. Our study underscores the need for adequate genetic profiling in melanoma patients. Personalized therapeutic strategies might provide more effective treatment solutions, enhancing survival rates and improving overall melanoma patient care.

## AUTHOR CONTRIBUTIONS

**Nilesh Kodali:** Conceptualization (lead); data curation (lead); formal analysis (equal); investigation (lead); methodology (lead); project administration (lead); resources (equal); software (equal); supervision (lead); validation (lead); visualization (lead); writing – original draft (lead); writing – review and editing (equal). **Simona Alomary:** Methodology (equal); resources (equal); validation (equal); visualization (equal); writing – original draft (equal); writing – review and editing (equal). **Abhijit Bhattaru:** Formal analysis (lead); methodology (lead); project administration (equal); validation (lead); visualization (lead); writing – review and editing (equal). **Ahmed Eldaboush:** Investigation (equal); validation (equal); writing – review and editing (equal). **Robert A. Schwartz:** Conceptualization (equal); investigation (equal); methodology (equal); project administration (equal); supervision (equal); validation (equal); visualization (equal); writing – review and editing (equal). **Shari R. Lipner:** Conceptualization (equal); investigation (equal); methodology (equal); project administration (equal); supervision (lead); validation (equal); visualization (equal); writing – review and editing (equal).

## FUNDING INFORMATION

The authors report no funding sources relevant to this work.

## CONFLICT OF INTEREST STATEMENT

Mr. Kodali, Ms. Alomary, and Mr. Bhattaru, Dr. Schwartz and Dr. Lipner have no conflicts of interest. Dr. Lipner has served as a consultant for Ortho‐Dermatologics, Lilly, Moberg Pharmaceuticals and BelleTorus Corporation.

## Supporting information


Data S1.

## Data Availability

The data that support the findings of this study are available from the corresponding author upon reasonable request.
