# Supplementary material for: Gender and melanoma subtype‐based prognostic implications of MUC16 and TTN co‐occurrent mutations in melanoma: A retrospective multi‐study analysis
Source: Cancer Med. 2024 Sep 6;13(17):e70199. doi: 10.1002/cam4.70199 (PMC11378355; doi:10.1002/cam4.70199)
Supplement: Supplementary file 1 — Data S1. [file CAM4-13-e70199-s001.docx]

**Supplemental Tables:**

**Table S1:** Mutation count, number of samples, and mutation frequency for the top 50 genes from total multi-study query.

| Gene | Mutation Count | Number of Samples | Mutation Frequency |
| --- | --- | --- | --- |
| *MUC16* | 3944 | 1433 | 62.90% |
| *TTN* | 5100 | 1606 | 61.10% |
| *BRAF* | 1181 | 2262 | 47.30% |
| *DNAH5* | 1627 | 1500 | 44.70% |
| *PCLO* | 1419 | 1433 | 40.80% |
| *CSMD1* | 943 | 1353 | 35.30% |
| *ADGRV1* | 871 | 1353 | 33.60% |
| *LRP1B* | 1073 | 1580 | 33.60% |
| *DNAH7* | 878 | 1433 | 31.30% |
| *ANK3* | 947 | 1500 | 30.60% |
| *APOB* | 832 | 1500 | 30.50% |
| *MGAM* | 654 | 1353 | 30.50% |
| *RP1* | 825 | 1500 | 30.30% |
| *XIRP2* | 712 | 1379 | 29.10% |
| *USH2A* | 738 | 1500 | 28.90% |
| *PTPRT* | 967 | 2115 | 28.70% |
| *PKHD1L1* | 796 | 1459 | 28.70% |
| *FLG* | 843 | 1500 | 28.60% |
| *MUC17* | 864 | 1580 | 28.60% |
| *DSCAM* | 630 | 1395 | 28.40% |
| *CSMD2* | 710 | 1500 | 28.10% |
| *TERT* | 762 | 2242 | 28.10% |
| *FAT4* | 793 | 1608 | 27.10% |
| *THSD7B* | 632 | 1433 | 26.70% |
| *DNAH9* | 653 | 1500 | 26.50% |
| *ZFHX4* | 587 | 1353 | 26.40% |
| *MXRA5* | 599 | 1500 | 26.30% |
| *DNAH8* | 813 | 1606 | 26.20% |
| *NRAS* | 596 | 2196 | 26.10% |
| *CSMD3* | 740 | 1580 | 26.00% |
| *FAT3* | 638 | 1433 | 26.00% |
| *HYDIN* | 637 | 1500 | 25.40% |
| *DNAH3* | 632 | 1500 | 25.20% |
| *PCDH15* | 592 | 1500 | 24.30% |
| *NEB* | 581 | 1433 | 24.30% |
| *OBSCN* | 545 | 1433 | 23.70% |
| *UNC13C* | 531 | 1379 | 23.50% |
| *TRA* | 114 | 346 | 23.40% |
| *GRIN2A* | 825 | 2342 | 23.10% |
| *WDFY4* | 117 | 330 | 22.70% |
| *SPHKAP* | 593 | 1580 | 22.70% |
| *MROH2B* | 426 | 1315 | 22.70% |
| *DCC* | 493 | 1500 | 22.30% |
| *PAPPA2* | 530 | 1433 | 22.10% |
| *WDR87* | 122 | 330 | 22.10% |
| *CACNA1E* | 492 | 1353 | 22.10% |
| *PREX2* | 683 | 2109 | 22.00% |
| *TENM3* | 457 | 1353 | 21.70% |
| *SCN10A* | 517 | 1500 | 21.70% |

|  |  |  |  |
| --- | --- | --- | --- |

**Table S2:** Co-occurrence analysis of top 50 genes from the total multi-study query.

| A | B | Neither | A Not B | B Not A | Both | Log2 Odds Ratio | P-Value | q-Value | Tendency |
| --- | --- | --- | --- | --- | --- | --- | --- | --- | --- |
| *MUC16* | *TTN* | 461 | 117 | 193 | 741 | >3 | <0.001 | <0.001 | Co-occurrence |
| *TTN* | *DNAH5* | 473 | 384 | 105 | 550 | 2.69 | <0.001 | <0.001 | Co-occurrence |
| *MUC16* | *DNAH5* | 526 | 331 | 128 | 527 | 2.71 | <0.001 | <0.001 | Co-occurrence |
| *TTN* | *BRAF* | 400 | 438 | 178 | 496 | 1.348 | <0.001 | <0.001 | Co-occurrence |
| *TTN* | *PCLO* | 510 | 440 | 68 | 494 | >3 | <0.001 | <0.001 | Co-occurrence |
| *MUC16* | *PCLO* | 577 | 373 | 77 | 485 | >3 | <0.001 | <0.001 | Co-occurrence |
| *MUC16* | *BRAF* | 458 | 380 | 196 | 478 | 1.556 | <0.001 | <0.001 | Co-occurrence |
| *TTN* | *LRP1B* | 520 | 471 | 58 | 463 | >3 | <0.001 | <0.001 | Co-occurrence |
| *MUC16* | *LRP1B* | 567 | 424 | 87 | 434 | 2.738 | <0.001 | <0.001 | Co-occurrence |
| *TTN* | *CSMD1* | 528 | 511 | 50 | 423 | >3 | <0.001 | <0.001 | Co-occurrence |
| *TTN* | *RP1* | 532 | 524 | 46 | 410 | >3 | <0.001 | <0.001 | Co-occurrence |
| *MUC16* | *CSMD1* | 584 | 455 | 70 | 403 | 2.885 | <0.001 | <0.001 | Co-occurrence |
| *TTN* | *ADGRV1* | 541 | 532 | 37 | 402 | >3 | <0.001 | <0.001 | Co-occurrence |
| *TTN* | *DNAH7* | 543 | 535 | 35 | 399 | >3 | <0.001 | <0.001 | Co-occurrence |
| *TTN* | *ANK3* | 523 | 536 | 55 | 398 | 2.82 | <0.001 | <0.001 | Co-occurrence |
| *MUC16* | *ADGRV1* | 609 | 464 | 45 | 394 | >3 | <0.001 | <0.001 | Co-occurrence |
| *TTN* | *DNAH8* | 530 | 544 | 48 | 390 | 2.985 | <0.001 | <0.001 | Co-occurrence |
| *TTN* | *PKHD1L1* | 527 | 544 | 51 | 390 | 2.889 | <0.001 | <0.001 | Co-occurrence |
| *TTN* | *APOB* | 522 | 545 | 56 | 389 | 2.734 | <0.001 | <0.001 | Co-occurrence |
| *TTN* | *MGAM* | 544 | 549 | 34 | 385 | >3 | <0.001 | <0.001 | Co-occurrence |
| *TTN* | *FLG* | 527 | 550 | 51 | 384 | 2.851 | <0.001 | <0.001 | Co-occurrence |
| *MUC16* | *RP1* | 581 | 475 | 73 | 383 | 2.682 | <0.001 | <0.001 | Co-occurrence |
| *MUC16* | *DNAH7* | 601 | 477 | 53 | 381 | >3 | <0.001 | <0.001 | Co-occurrence |
| *TTN* | *MUC17* | 508 | 555 | 70 | 379 | 2.309 | <0.001 | <0.001 | Co-occurrence |
| *MUC16* | *PKHD1L1* | 590 | 481 | 64 | 377 | 2.853 | <0.001 | <0.001 | Co-occurrence |
| *MUC16* | *ANK3* | 576 | 483 | 78 | 375 | 2.519 | <0.001 | <0.001 | Co-occurrence |
| *TTN* | *FAT4* | 544 | 561 | 34 | 373 | >3 | <0.001 | <0.001 | Co-occurrence |
| *DNAH5* | *PCLO* | 667 | 283 | 190 | 372 | 2.206 | <0.001 | <0.001 | Co-occurrence |
| *MUC16* | *FLG* | 590 | 487 | 64 | 371 | 2.812 | <0.001 | <0.001 | Co-occurrence |
| *TTN* | *CSMD3* | 519 | 563 | 59 | 371 | 2.535 | <0.001 | <0.001 | Co-occurrence |
| *TTN* | *USH2A* | 522 | 564 | 56 | 370 | 2.612 | <0.001 | <0.001 | Co-occurrence |
| *MUC16* | *MUC17* | 572 | 491 | 82 | 367 | 2.382 | <0.001 | <0.001 | Co-occurrence |
| *MUC16* | *DNAH8* | 582 | 492 | 72 | 366 | 2.588 | <0.001 | <0.001 | Co-occurrence |
| *DNAH5* | *LRP1B* | 698 | 293 | 159 | 362 | 2.439 | <0.001 | <0.001 | Co-occurrence |
| *MUC16* | *TTN* | 461 | 117 | 193 | 741 | >3 | <0.001 | <0.001 | Co-occurrence |
| *TTN* | *DNAH5* | 473 | 384 | 105 | 550 | 2.69 | <0.001 | <0.001 | Co-occurrence |
| *MUC16* | *DNAH5* | 526 | 331 | 128 | 527 | 2.71 | <0.001 | <0.001 | Co-occurrence |
| *TTN* | *BRAF* | 400 | 438 | 178 | 496 | 1.348 | <0.001 | <0.001 | Co-occurrence |
| *TTN* | *PCLO* | 510 | 440 | 68 | 494 | >3 | <0.001 | <0.001 | Co-occurrence |
| *MUC16* | *PCLO* | 577 | 373 | 77 | 485 | >3 | <0.001 | <0.001 | Co-occurrence |
| *MUC16* | *BRAF* | 458 | 380 | 196 | 478 | 1.556 | <0.001 | <0.001 | Co-occurrence |
| *TTN* | *LRP1B* | 520 | 471 | 58 | 463 | >3 | <0.001 | <0.001 | Co-occurrence |
| *MUC16* | *LRP1B* | 567 | 424 | 87 | 434 | 2.738 | <0.001 | <0.001 | Co-occurrence |
| *TTN* | *CSMD1* | 528 | 511 | 50 | 423 | >3 | <0.001 | <0.001 | Co-occurrence |
| *TTN* | *RP1* | 532 | 524 | 46 | 410 | >3 | <0.001 | <0.001 | Co-occurrence |
| *MUC16* | *CSMD1* | 584 | 455 | 70 | 403 | 2.885 | <0.001 | <0.001 | Co-occurrence |
| *TTN* | *ADGRV1* | 541 | 532 | 37 | 402 | >3 | <0.001 | <0.001 | Co-occurrence |
| *TTN* | *DNAH7* | 543 | 535 | 35 | 399 | >3 | <0.001 | <0.001 | Co-occurrence |
| *TTN* | *ANK3* | 523 | 536 | 55 | 398 | 2.82 | <0.001 | <0.001 | Co-occurrence |
| *MUC16* | *ADGRV1* | 609 | 464 | 45 | 394 | >3 | <0.001 | <0.001 | Co-occurrence |
